# Studies of Health Effects from Nuclear Testing near the Semipalatinsk Nuclear Test Site, Kazakhstan

**DOI:** 10.5195/cajgh.2015.127

**Published:** 2015-05-08

**Authors:** Bernd Grosche, Tamara Zhunussova, Kazbek Apsalikov, Ausrele Kesminiene

**Affiliations:** 1 Federal Office for Radiation Protection, Department of Radiation Protection and Health, Oberschleissheim, Germany;; 2 Norwegian Radiation Protection Authority, Department of Monitoring and Research, Osteras, Norway;; 3 Scientific Research Institute for Radiation Medicine and Ecology, Semey, Kazakhstan;; 4 International Agency for Research on Cancer, Lyon, France

**Keywords:** Semipalantinsk nuclear test site, Kazakhstan, radiation health, environmental health

## Abstract

The nuclear bomb testing conducted at the Semipalatinsk nuclear test site in Kazakhstan is of great importance for today’s radiation protection research, particularly in the area of low dose exposures. This type of radiation is of particular interest due to the lack of research in this field and how it impacts population health. In order to understand the possible health effects of nuclear bomb testing, it is important to determine what studies have been conducted on the effects of low dose exposure and dosimetry, and evaluate new epidemiologic data and biological material collected from populations living in proximity to the test site. With time, new epidemiological data has been made available, and it is possible that these data may be linked to biological samples. Next to linking existing and newly available data to examine health effects, the existing dosimetry system needs to be expanded and further developed to include residential areas, which have not yet been taken into account. The aim of this paper is to provide an overview of previous studies evaluating the health effects of nuclear testing, including some information on dosimetry efforts, and pointing out directions for future epidemiologic studies.

The Semipalatinsk nuclear test site (STS) is located in the present East-Kazakhstan Oblast (or administrative division) of Kazakhstan, a country that was previously a part of the Soviet Union. The test site is named after the city of Semipalatinsk (in Kazakh: Semey) and is located approximately 150 km west of the city. The test site covers an area of 18,500 km^2^, or 7,143 square-miles. The STS was a major site for nuclear weapons testing by the former Soviet Union, and it was where the former Soviet Union conducted their first nuclear test on August 29, 1949. Afterwards, 456 nuclear explosions were carried out between 1949 and 1989, including 111 atmospheric events (86 air events and 25 surface events) between 1949 and 1962.^1^,^2^ After the Limited Test Ban Treaty was signed in 1963, the tests at STS were restricted to underground shafts and tunnels so that little or no offsite environmental contamination was caused; except for four events that occured between 1965 and 1968, within the framework of peaceful uses of nuclear energy program, which was designed for earth-moving purposes (e.g. the construction of artificial lakes, canals, and harbors). The last event conducted at the STS was on October 19, 1989. The total yield of atmospheric events conducted at the STS is reported to be 6.58 megatons of TNT equivalent, which corresponds to approximately 66% of the total estimated Soviet bomb yield.^3^

The test site had three major testing areas. Atmospheric bomb tests were performed at Ground Zero. Over 200 underground nuclear tests were performed in the Degelen Mountains. 123 underground nuclear tests were conducted in the Balapan area, one of which led to the formation of Lake Chagan (or Lake Balapan), which is sometimes called ‘Atomic Lake’ due to the current radioactive environment.^2^ During the tests, access to the site was strictly controlled by the Soviet armed forces, and no civilian use of the area was permitted.

Previous analyses made by several institutes from different countries might provide insight into the health effects from low dose radiation exposure, as defined by the European low-dose initiative MELODI.^4^,^5^ There have also been numerous biological studies published on radiation effects.^6^–^11^ The European Commission has funded a new project to assess whether it is possible to create a unified cohort of individuals affected by the atomic bomb testing,^12^ which, if successful, could provide numerous insights into the health effects of radiation exposure. Beyond giving an overview on already published evidence, this paper provides recommendations about future research directions on radiation health effects. In particular, the authors would like to argue that it is necessary to better link future epidemiological cohort studies with biological research either based on already collected, existing biomaterial or on biomaterial that will be sampled in the frame of the future investigations. This manuscript aims to provide an overview on previous studies focused on the health effects of the nuclear testing by evaluating information on dosimetry efforts and insights on the direction of future epidemiological studies.

## Environmental health exposures

The 111 atmospheric events conducted between 1949 and 1962 caused the primary source of radioactive contamination of the environment and the majority of the radiation exposure of the public. The most damaging tests, in terms of exposure, were those conducted on August 29, 1949 (with a yield of 22 kilotons (kt) TNT equivalent), September 24, 1951 (38 kt), August 12, 1953 (400 kt), and August 24, 1956 (27 kt). Most of the other explosions led to exposures that affected only the test ground, not the vicinity of the test site.

The population living closest to the test site was exposed to relatively high levels of radiation. Settlements affected by the 1949 test were located north-east of the test site (e.g. Dolon and Cheremushka), but traces from this test have also been documented in residents living further away in the Altai region in Russia.^13^ The tests of 1951, 1953, and 1956 affected settlements south and south-east of the test site (e.g. Kainar, Karaul, Kaskabulak, Sarzhal, and Znamenka).

Both, external and internal exposures need to be considered when estimating the individual doses to which residents have been exposed. External exposure was caused by the radioactive clouds and submersion, whereas internal exposure is caused by the consumption of contaminated foods. Food consumption varied between the different ethnic groups living in the contaminated areas, such as Kazakhs, Russians, and Germans. The first effort to evaluate both external and internal exposure was attempted in Kazakhstan and published in 2000.^14^ The extensive work of different international groups has found that the calculated doses tended to overestimate the actual exposures,^15^–^18^ where the village of Dolon was used as the primary location for comparing different methods for dose estimates. During a research meeting held at the University of Hiroshima in 2005, researchers with different approaches to retrospective dosimetry compared and discussed their results, paying close attention to reasons for large variation among estimates. The results from this workshop were published in a special issue of the Japanese Journal for Radiation Research in 2006, with agreement achieved about exposure doses for Dolon.^19^ Today, the previously published U.S./Russian joint methodology is considered as being the most appropriate.^18^

### Previous studies on health effects

The first studies on health effects associated with nuclear bomb testing were of a descriptive nature. One of the first published reports focused on the increased age-specific incidence rates of malignant tumors in the Semipalatinsk Oblast compared to the general population of the Soviet Union and Kazakhstan populations.^20^ Based on the survey data that was collected in five year intervals from 1949 onwards, increased cancer incidence rates were reported in highly exposed villages compared to the villages of the Kokpektinskii district (control area).^21^ In an ecological study of childhood cancer incidence in four administrative divisions adjacent to the STS spanning from 1981 to 1990, an increase in relative risks for all cancers, including leukemia and brain tumors, were reported in children living less than 200 km from the test epicenter compared to children residing more than 400 km from the test site.^22^ In 2000, a comprehensive book was published, which included information on the incidence and mortality of a few selected diseases such as cancer, cardiovascular disease, and mental retardation, as well as the prevalence of congenital malformations.^2^ Overall, there was clear evidence of unfavorable health outcomes in the population living around the test site.

**Figure 1:**
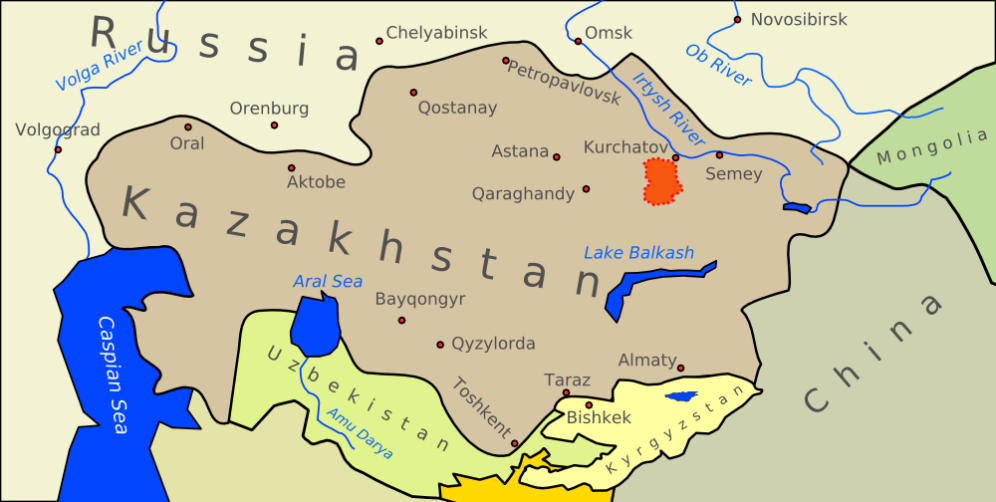
**The 18,000 km^2^ area of the Semipalantinsk Test Site (indicated in red attached to Kurchatov (along the Irtysh river), and near Semey, as well as Karaganda and Astana**

Based on data that were collected from 1960 to 1991 by the National Research Institute of Radiation Medicine and Ecology of Kazakhstan, a cohort was established which became known as the “historical” cohort. This cohort was comprised of approximately 20,000 individuals. Half of these individuals came from exposed villages and the other half came from a comparison (unexposed) area. The first analyses were based on the dosimetry system developed by Kurakina et al.^14^ and focused on cancer mortality with follow-ups until 1999.^23^,^24^ A more recent analysis employed a dosimetry system, which was developed by the U.S. National Cancer Institute for the purpose of studying thyroid diseases, amongst the population living near the Semipalatinsk nuclear test site (see Figure 1).

The dosimetry system was based on a joint U.S./Russian dose reconstruction methodology that combined the experience of dose-reconstruction scientists in Russia and the U.S.^16^,^18^ Another study looked at mortality associated with cardiovascular disease.^25^ While given doses, around 90 milliGray (mGy) on average, with a maximum of 630 mGy, had no effect on cardiovascular disease, the analyses of mortality due to solid tumors had to be revised to incorporate the updated dosimetry system. The dosimetry system developed by Kurakina et al.^14^ generally gave higher dose estimates in comparison to more recent systems,^16^,^18^ including the University of Hiroshima consensus meeting.

A cross-sectional study of the prevalence of thyroid diseases was conducted using 2,994 residents from eight villages. The study involved ultrasound screening, and malignancy status was confirmed by cytopathology evaluation. In terms of excess relative risk per unit dose, the dose-response findings for nodule prevalence were compatible with those from populations exposed to medical X-rays and atomic bomb survivors.^26^ Another study looking at the differences between birth cohorts of those in East Kazakhstan found that those born closest to the dates during the nuclear tests had an increased risk of numerous health problems, including cardiocascular disease and cancer.^27^

Two studies looked into possible radiation effects in the offspring of exposed individuals. The first study focused on investigating whether the exposures led to a significant change in the sex ratio of newborns. Based on 11,464 single births from 3,992 mothers exposed to radiation during 1949–1956, the overall sex ratio was 1.07 (e.g. 107 boys per 100 girls), which was comparable to the sex ratio in Kazakhstan in the mid-2000s (1.06).^28^ Using a subset of 141 twin deliveries from 3,992 mothers, further analyses were conducted on the effects of radiation exposure on same sex and different sex twin delivery. There was an increase in the odds of having different sex twins for births occurring within 5 years after exposure compared with more than 20 years after exposure [OR = 4.08 (95% CI: 1.11, 15.07)] in all villages, regardless of exposure level.^29^ Another study focused on the frequency of mini-satellite mutations in exposed offspring and unexposed (control) offspring, and found a negative correlation between mutation rate and the parental year of birth in the exposed F1 generation, with the highest mutation rate in the most exposed cohort of parents born before 1960.^8^

To our knowledge, no additional studies have been conducted on mental disability or congenital malformation.

### Data bases

Several databases and registries have been developed over the past few decades, which need to be further explored for risk analyses. One of these resources is the previously mentioned historical cohort.^23^ Another database is the registry of the population of the former Semipalatinsk Oblast. Initiated in 1949, this registry is a valuable source of health data, including information on residential histories, vital statistics, and causes of death for over 100,000 individuals.^30^ This database includes the participants of the historical cohort. More recently, a joint Japanese-Kazakh effort was initiated by a group headed by the late Dr. Ogiu from Japan, focusing on creating a database of exposed residents and those from a comparison area – which is different from the one used for the historical cohort. This database was designed to include residential history and causes of death.^31^ As mentioned earlier, one of the tasks of the SEMI-NUC project^12^ is to test how the two databases may be linked. A respective report is under development, which will define how to best use available data from both databases for future epidemiological research.

### Biological material

Biological samples from individuals living in the affected areas have been collected in numerous studies;^6^–^11^ however, to our knowledge, not all were stored. In recent years, the National Research Institute of Radiation Medicine and Ecology in Semey started collecting blood samples and teeth from the persons still residing in the villages close to the former STS and stored them in a biobank.^32^

## Discussion

This review demonstrates that different datasets are available for investigating health effects of radiation among the population living in proximity to the Semipalatinsk nuclear testing site. The EU-funded project SEMI-NUC aims to evaluate to what extent the different data sets can be linked and whether there is a possibility to use this data for a future prospective cohort study. To that end, possible follow-up mechanisms are going to be tested, including monitoring of incidence and mortality data, where the outcomes of interest are cancer and non-cancerous diseases. Avenues of ascertainment of vital statistics and cause of death have yet to be identified and validated. Thus, available sources of information must be defined (e.g. death certificates, hospital records, etc.). Methodologies that were used to calculate doses for existing cohorts have to be reviewed and compared, including identifying potential discrepancies and reasons for the discrepancies. Furthermore, it will be important to evaluate the quality and the content of existing biomaterials. Such evaluations will need to look at how samples were processed and stored, which types of equipment was used for DNA extraction, methods of evaluation of previous cytogentic analyses, and the condition of stored tissue samples. In addition, there will be evaluations of the possible use for modern technology analysis. For the latter, validated SOPs have already been developed and are available on the internet.^33^ Newly collected biomaterial will mainly come from elderly participants since the last bomb testing that led to significant exposures to the public was conducted in 1954.

However, there are other interesting options for future research on the effects of low to intermediate doses. The large data sets independently developed by Katayama et al.^30^ and researchers from the Radiation Effects Association^31^ give rise to the possibility of investigating transgenerational effects. Further, there is a three-generation data set available at NIIRME, which includes limited results from previously conducted chromosomal aberration analyses. The collection of the relevant information for this dataset is currently underway.

Although research on the health effects of low to medium exposures from the nuclear testing is of high public health relevance, a major limitation is that studies mentioned in this paper are not well connected with each other. This makes an overall joint evaluation difficult. Additionally, epidemiological research in the area close to the test site faces a number of difficulties. One of the problems is that exposures are dominated by the radioactive plumes (external), but for some health endpoints, internal exposures are of a higher relevance. Since exposure estimates have been carried out many years after actual events, this introduces another source of bias. Another limitation is that biological materials from the affected population were not always properly stored and labeled. It is difficult to link available biological material to individuals included in the epidemiological studies and their demographic data.

Despite the fact that difficulties exist, studies of individuals affected by atomic bomb testing have the potential to contribute to a better understanding of radiation exposure risk, particularly because the population is not a randomly selected one (e.g. nuclear workers have much fewer and lower exposures in comparison to atomic bomb survivors).^34^,^35^ However, calculating internal exposures is still a challenge. For a complete coverage of the area affected by the nuclear bomb testing at the Semipalatinsk test site, dose estimates have to be developed for settlements that have not yet been included in the existing dosimetry systems. Accordingly, one of the tasks of the SEMI-NUC project is to test the feasibility of dose reconstruction.

In summary, data are available for more than 100,000 persons forming a large cohort which needs to be further investigated.^30^ Furthermore, the range of external doses as described in the study of cardiovascular diseases (i.e. 0–630 mGy) is wide enough to conduct meaningful health studies.^25^ Lastly, the data from the 3-generation studies are of high interest to study transgenerational effects. Overall, this line of research has great relevance not only for the region of Central Asia but also to countries around the world affected by nuclear testing.
